# Evaluation of the Validity and Reliability of NeuroSkin’s Wearable Sensor Gait Analysis Device in Healthy Individuals

**DOI:** 10.3390/bioengineering12090960

**Published:** 2025-09-06

**Authors:** Maël Descollonges, Baptiste Moreau, Nicolas Feppon, Oussama Abdoun, Perrine Séguin, Lana Popovic-Maneski, Julie Di Marco, Amine Metani

**Affiliations:** 1 Kurage, 69100 Villeurbanne, France; baptiste.moreau@kurage.fr (B.M.); nicolas.feppon@kurage.fr (N.F.); perrine.seguin@kurage.fr (P.S.); amine.metani@kurage.fr (A.M.); 2 SMR Val Rosay, 69370 Saint-Didier au Mont d’Or, France; dr.jdimarco@gmail.com; 3 Laboratoire Images, Signaux et Systèmes Intelligents (LISSI), 94400 Vitry sur Seine, France; 4 Independent Researcher; 5 Institute of Technical Sciences of SASA, 35/IV 11000 Belgrade, Serbia; 6 Laboratoire de Physique, Université de Lyon, Ecole Normale Supérieure de Lyon (ENS Lyon), Centre National de la Recherche Acientifique (CNRS), 69007 Lyon, France

**Keywords:** quantitative gait analysis (QGA), wearable sensors, embedded sensors, GAITRite, NeuroSkin

## Abstract

Gait analysis plays a crucial role in assessing and monitoring the progress of individuals undergoing rehabilitation. This preliminary validation study aims to compare the performance of a new wearable system, NeuroSkin^®^, equipped with embedded sensors (inertial measurement unit and pressure sensors), with the non-wearable gold standard, GAITRite^®^, in assessing spatio-temporal parameters during gait. Data was collected from nine healthy participants wearing the NeuroSkin while walking on the GAITRite walkway. Temporal parameters were calculated using the pressure sensors of the NeuroSkin^®^ to detect heel strike (HS) and toe off (TO) on both sides. Distances were calculated using vertical hip acceleration with an inverted pendulum method. We found that the level of agreement between NeuroSkin^®^ and GAITRite^®^ measures was excellent for speed, cadence, as well as length and duration of stride and step (lower bound of intraclass correlation coefficients (ICCs) > 0.95), and moderate to excellent for stance and swing durations (ICC > 0.5). These levels of agreement are comparable to the known test–retest reliability of GAITRite^®^ measures. These results demonstrate the potential of NeuroSkin^®^ as an embedded gait assessment system for healthy subjects. As this study was conducted exclusively in healthy adults, the results are not directly generalizable to clinical populations. Thus, future studies are needed to investigate its use in patients.

## 1. Introduction

Assessing gait using either wearable or non-wearable systems in individuals is a widespread practice in research and clinical settings [1]. Spatiotemporal gait parameters are commonly measured by clinicians to diagnose gait disorders and monitor disease progression [2]. The GAITRite^®^ gait analysis system has long been recognized as a gold standard for measuring and analyzing gait parameters [3]. Its validity and reliability have been well-documented in healthy individuals [3,4,5]. However, non-wearable systems such as GAITRite^®^ are restricted to controlled research environments and do not allow gait analysis during regular daily activities [1,6]. For example, the use of the GAITRite^®^ mat requires significant space and is cumbersome to manage [7]. These limitations may hinder a comprehensive assessment of patients with gait disorders, highlighting the need for user-friendly devices that can be integrated into both outpatient and clinical routines, therefore enhancing the monitoring of disability progression. Inertial sensors—combining accelerometers and gyroscopes—may offer a wearable alternative, and the validity of such systems is well-documented [7,8,9,10,11,12,13]. Wearable gait analysis systems are increasingly being explored across diverse populations, including pediatric cohorts, where they allow early detection and intervention in developmental or neurological disorders [14]. This demonstrates the growing relevance of wearable-based methodologies beyond young and adult populations. NeuroSkin^®^ (Kurage, Lyon, France) [15,16] is a class IIa medical device, combining multi-channel functional electrical stimulation (MFES) with a wearable gait analysis system that relies on embedded inertial measurement units (IMUs) and foot pressure sensors. It is designed for the gait rehabilitation of patients with neurological motor disorders (such as post-stroke, spinal cord injury, or multiple sclerosis patients). To date, few gait assistance devices integrate MFES-walking [16], and even fewer are equipped with sensors capable of detecting gait phases, triggering stimulation in a synchronized manner: None of these devices currently offer integrated quantitative gait analysis (QGA). Therefore, this proof-of-concept study aimed to assess how NeuroSkin compares to a clinically widespread such as GAITRite, according to spatiotemporal gait parameters in healthy adults.

## 2. Materials and Methods

### 2.1. Data Collection

The data were collected on nine healthy subjects (6 males; 3 females; mean age, 37.5 years old; mean height, 175 ± 6.5 cm; mean weight, 75 kg) wearing NeuroSkin^®^ while walking in a straight line on the GAITRite^®^ walkway. Subjects were healthy and free of any musculoskeletal or neurological conditions that could affect their gait. The data were collected and processed in compliance with the General Data Protection Regulation (GDPR), ensuring the protection of individuals’ privacy and personal data. All subjects were informed that the data obtained from the sensors embedded in NeuroSkin^®^ and GAITRite^®^ systems would be used for research.

### 2.2. Gait Analysis Systems

**Figure 1:**
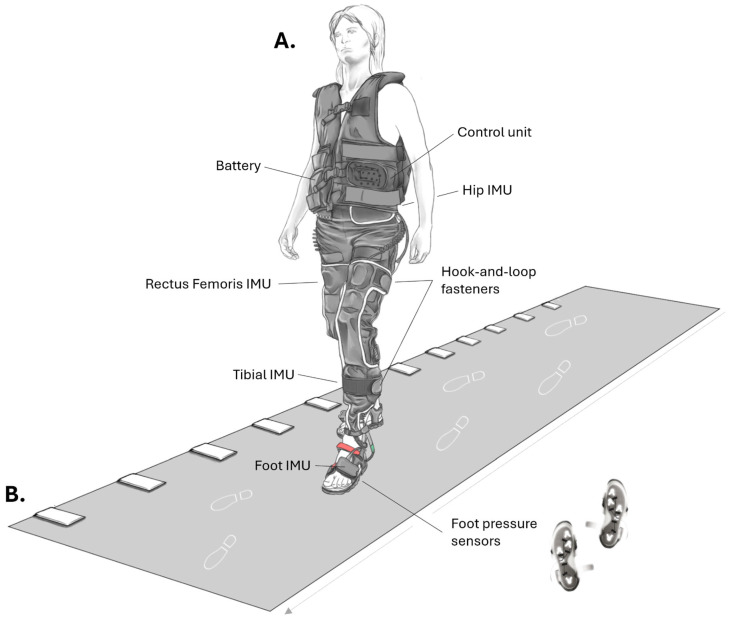
Gait analysis systems used. (**A**) NeuroSkin^®^ and (**B**) GAITRite^®^ systems. IMU: Inertial Measurement Unit. The device includes fitted pants with IMU sensors on the hip (back part), legs (over the rectus femoris muscle and tibia bone), and feet (dorsal part), and pressure-sensing insoles. A vest on the torso contains the data acquisition unit and a gateway for uploading raw data to the Cloud for further processing and analysis. The same experimenter placed the IMUs on the dedicated attachment points. The system captures gait signals at 100 Hz alongside GAITRite^®^ measurements.

Subjects walked on the GAITRite^®^ system, which is a pressure-sensitive roll-up walkway [3], while simultaneously wearing the NeuroSkin^®^ system [16] (Figure 1). The GAITRite^®^ system was installed in a quiet and dimly lit room and connected to a computer running the 4.8.7 GAITRite^®^ software (CIR Systems Inc., Clifton, NJ, USA), which recorded temporal and spatial gait parameters. The GAITRite^®^ walkway system measures 7 × 0.9 m with an active sensor area of 6.1 × 0.6 m.

The NeuroSkin^®^ device (NeuroSkin^®^, Kurage, France) takes the form of tight-fitting pants worn by participants during walking. It consists of seven inertial measurement units (IMUs) placed on the back hip (5 × 6.9 × 2.8 cm), upper leg (over the Rectus Femoris), lower leg (on the Tibia), and dorsal side of the feet (4 × 7 × 1.3 cm), along with four pressure sensors embedded in the sandals insoles and positioned under the heel, first and fifth metatarsals, and toe regions (Figure 1). NeuroSkin sandals are modified sandals (Quechua sandals, Decathlon, France). Sensors were positioned by the same experimenter. The IMUs are not integrated into the pants themselves but are embedded in an additional textile layer attached with hook-and-loop fasteners to minimize placement variability (Figure 1). A body-worn vest houses a microcontroller platform at the front (control unit), which records data and runs gait analysis algorithms, and the stimulator and lower back IMU at the back. The complete system weighs 3.925 kg. GAITRite^®^ and NeuroSkin^®^ systems simultaneously recorded data at a sampling rate of 100 Hz.

### 2.3. Walking Test Procedure and Measurements

The experiment was conducted in a single session, in which each participant performed one walking trial on the GAITRite^®^ system while wearing the NeuroSkin^®^ system. The GAITRite^®^ was installed in a quiet and spacious room, allowing the participant to start walking approximately two meters before reaching the mat and to continue walking for two meters beyond the end of the mat without slowing down. This setup ensures that the participant maintains a stable walking speed across the active sensor area [3]. Participants were instructed to walk at a moderate and comfortable pace (Table 1). Before the main walking trial, each participant performed a familiarization trial to verify that all sensors were properly fixed and to become accustomed to wearing the NeuroSkin^®^ system. Following this familiarization, participants stood still for 30 s with their feet shoulder-width apart to allow the system to stabilize and calibrate the sensors. After this calibration period, the main walking trial was conducted. Gait parameters were recorded simultaneously by both systems for analysis and comparison.

Temporal parameters, including stride, step, swing, and stance times (in seconds) as well as cadence, were computed using NeuroSkin’s sandals pressure sensors (Kurage, Lyon, France). Gait events (heel strike (HS) and toe-off (TO)) were detected on both sides using a threshold-based method based on the heel pressure sensor for heel strike and the sum of the metatarsal and toe pressure sensors for toe-off detection. Stance and swing phases were defined based on these events. Spatial parameters (step length and stride lengths) and speed were calculated using hip vertical acceleration with an inverted pendulum method [17]: Vertical acceleration is acquired using the gravity axis, low-pass Butterworth filtered at 20 Hz (order 4), and integrated twice to get the vertical displacement, from which the step length can be calculated. In this pendulum model, changes in the height of the center of movement depend on step length. Therefore, when changes in height are known, step length can be predicted from geometrical characteristics as follows:

step length = 2√2lh − h^2^

### 2.4. Statistical Analysis

The following statistical analysis was designed to evaluate the reliability of the NeuroSkin^®^ embedded sensor system. Data were first screened for normality of distribution using the Shapiro–Wilk test. The values for each leg (left and right) were averaged for each parameter (Table 2). Then, a leg-by-leg unilateral analysis was performed (Table 3), since results may vary between legs in patients with neurological disorders. This supplementary analysis provides valuable insights into potential leg-specific differences and lays the foundation for future research in this area.

To comply with the methodology used in previous studies assessing the level of agreement between two gait sensor systems [18,19], the concurrent validity of the NeuroSkin^®^ sensor system with the GAITRite^®^ device on outcome measures was also assessed using the intraclass correlation coefficient (ICC). We used the ICC (3,1), based on a two-way mixed effect model that assumes that raters (here, the sensor systems) are fixed, i.e., the only ones of interest, and the “absolute agreement” definition [20], as implemented in the R package irr (version 0.84.1). The ICC values were interpreted using the guidelines of Koo & Li [20]: values less than 0.5 are indicative of poor reliability, values between 0.5 and 0.75 indicate moderate reliability, values between 0.75 and 0.9 indicate good reliability, and values greater than 0.90 indicate excellent reliability. A sample size of 9 participants is sufficient to detect a correlation greater than 0.9 with 95% probability, which supports the adequacy of our sample size for assessing the concordance between measurements of the two devices.

In addition, the minimal detectable change with 95% confidence (MDC_95_) was calculated using the following formula:

$$MDC_{95}=1.96\;\times SD_{pooled}\;\times \sqrt{1-r}$$

where *r* is the correlation across the group between the NeuroSkin and GAITRite measures.

Furthermore, to provide a measure of accuracy, we calculated the absolute relative error (ARE) as the absolute difference between the measures of the two sensor systems, divided by the measure of the GAITRite. The AREs were calculated for each participant, and the median and 95% confidence intervals (CI) were estimated using the bias-corrected and accelerated bootstrap (BCa) algorithm implemented in the R package car (version 3.1.2). ARE is expressed as a percentage (Table 2 and Table 3).

## 3. Results

Table 2 and Table 3 present the descriptive statistics (means ± standard deviations) and the results of ICC and ARE for both legs’ average and by leg, respectively. The level of agreement between the NeuroSkin^®^ and the GAITRite^®^ measures was excellent for speed and cadence, as well as the length and duration of stride and step (lower bound of the intraclass correlation coefficients > 0.95), and moderate to excellent for stance and swing durations.

Table 2 also presents the minimal detectable changes (MDC_95_) and the signed relative error (SRE) for the average of both legs. The SRE indicates that there is no systematic bias of NeuroSkin measures compared to the GAITRite ones, except for a small overestimation of stride and step time (<1%) and stance time (<4%). Interestingly, the MDC_95_ between the two devices does not exceed 13 ms and 3 cm for stride duration and stride length, respectively. These values are well below the MDC_95_ estimated by Parati et al. [21] from 39 studies assessing the test–retest reliability of the GAITRite system (100 ms and 10 cm for healthy adults, respectively).

## 4. Discussion

In the present study, a wearable QGA device (NeuroSkin^®^) was used to provide a comprehensive assessment of healthy participants’ gait parameters. This study aimed to compare the temporal and spatial gait parameters computed by a system considered the gold standard (GAITRite^®^) [3] and the novel NeuroSkin^®^ system. Statistical analysis showed that gait parameters are similar for both systems, as evidenced by the moderate to excellent level of agreement between the NeuroSkin^®^ and GAITRite^®^ measurements reported by the intraclass correlation coefficient (ICC). This suggests that NeuroSkin^®^ is a reliable and valid tool for assessing gait quality in healthy individuals. These results are consistent with previous studies, which also compared wearable systems with the non-wearable GAITRite^®^ analysis system [7,19]. Byun et al. [19] and Vítečková et al. [7] both reported excellent agreement for basic gait parameters but highlighted lower reliability for gait variability and phase-specific measures (stance, swing, double support). Similarly, NeuroSkin^®^ showed strong agreement for global spatiotemporal metrics, while stance and swing times were more variable, underscoring the need for cautious interpretation in clinical populations. Stance and swing times parameters are potentially influenced by shoe sole properties, foot movements within the shoe, or possible misalignment of the sensors under the heel and toe areas. However, all participants wore appropriately sized shoes, minimizing this potential source of variability.

While these results appear promising, this study still has some limitations. Although participants underwent familiarization tests, only a single trial was considered for analysis. While it would be expected that more trials would enhance the robustness and reliability of the results, a previous report has shown that the plateau of test–retest reliability can be reached with as few as 8 steps [19]. Although the GAITRite system is considered a gold standard for gait analysis, it would be valuable and interesting to evaluate the accuracy of NeuroSkin’s parameters against optical motion capture systems and conventional gait analysis platforms. Thus, incorporating optical motion capture or other gold-standard methods in future studies could provide more comprehensive validation, enabling detailed assessment of kinematics and identification of potential sources of error. Moreover, it is well known that gait speed is a key parameter in locomotor analysis, both under normal and pathological conditions [22]. Gait speed serves as a relevant reference point for identifying and quantifying gait alterations. The use of a single gait speed in the present study limits the generalizability of our findings to other walking conditions, which may influence the applicability of the results to real-world settings. In this regard, it would have been relevant to investigate different speed modulation scenarios, such as slow, self-selected, or comfortable, and fast walking speeds [22]. This approach is commonly used in studies involving aging populations or individuals with neurological conditions, as it allows for a more comprehensive assessment of locomotor flexibility and the adaptive strategies employed by participants. Including such conditions in future investigations would allow us to assess the robustness of measured parameters across a wider range of locomotor demands and to extend the validation to more ecological, real-world walking conditions (e.g., uneven surfaces, directional changes, environmental distractions), which may substantially affect spatiotemporal parameters and sensor accuracy. Moreover, we anticipate that the generalizability of our findings to larger sample sizes, whether through repeated trials or an increased number of participants, will vary depending on the parameter. For those demonstrating excellent inter-device agreement and narrow confidence intervals, our results are expected to be robust and replicable. In contrast, for parameters such as swing and stance times, which exhibited notably wide confidence intervals around the ICC, we acknowledge the limitations of our current findings. This highlights that caution should be exercised when extrapolating these results, particularly for parameters with lower inter-device agreement. A larger-scale study will be necessary to more accurately determine the level of agreement between the two systems for these specific measures. Furthermore, future research should include patients with neurological impairments (e.g., due to various pathologies and conditions such as stroke, multiple sclerosis, etc.), since their gait patterns substantially differ from those of healthy participants. Therefore, the current findings should not be assumed to directly apply to clinical populations, and interpretation in such contexts must be made cautiously. Lastly, while NeuroSkin’s system includes multiple sensors (IMUs and pressure insoles), we acknowledge that small variations in sensor placement may influence the accuracy of the measured spatiotemporal gait parameters. In the present study, sensors were carefully positioned for each participant, and the IMUs were generally very well secured to the pants, resulting in minimal placement variability. As previously mentioned, only the parameters related to the pressure sensors may be influenced by the properties of the shoe sole, foot movements within the shoe, or possible misalignment of the sensors under the heel and toe areas, although the shoe size was appropriate for each participant. Thus, the methods used in our study are designed to be relatively robust to such variations; however, we did not directly assess or quantify the impact of sensor placement variability on the calculated gait parameters. Moreover, it should be noted that the inverted pendulum model used in our study is known to provide gross estimates of step length and speed for older adults, pathological populations, or for non-straight walking [23], and should be compared in the future with other methods in these populations.

In conclusion, the results obtained from the comparative analysis of the NeuroSkin^®^ and GAITRite^®^ devices provide insights into embedded gait assessment tools. NeuroSkin^®^, with its embedded sensors, demonstrated comparable performance to the GAITRite^®^ system in measuring spatiotemporal parameters. Although this study was conducted in healthy adults, the ability of NeuroSkin^®^ to provide real-time, continuous gait data suggests potential applicability in neurological rehabilitation contexts. While we did not assess patients in this work, prior research has shown that technology-assisted gait interventions, such as virtual reality training post-stroke and rhythmic auditory cueing post-stroke or in aging populations [24,25,26], can meaningfully improve walking performance. These findings highlight how wearable gait monitoring devices like NeuroSkin^®^ could, in future studies, be integrated with evidence-based rehabilitation protocols to enhance sensory feedback and gait training. In line with this perspective, a recent multicenter feasibility study using NeuroSkin^®^ combined with functional electrical stimulation in stroke patients further supports the potential of such AI-driven wearable systems for gait recovery [16]. Overall, the portability, ease of use, and real-time data collection of NeuroSkin^®^ make it a promising tool for ecological gait analysis. Further research in clinical populations is needed to confirm the utility and reliability in rehabilitation and daily life contexts.

## Figures and Tables

**Table 1:** Step count, distance, and ambulation time values per participant.

	GAITRite & NeuroSkin		
Participants (P)	Step Count	Distance (cm)	Ambulation Time (Seconds)
P1	8	531.6	5.66
P2	7	512.8	3.82
P3	7	488.7	3.90
P4	6	485.7	3.25
P5	7	501.6	3.94
P6	7	581.4	4.45
P7	9	559.4	5.08
P8	8	556.3	4.82
P9	9	573.2	5.60
**Total**	**7.5 ± 1**	**532.3 ± 36.7**	**4.50 ± 0.84**

**Table 2:** Descriptive statistics of data analyzed from both legs were averaged. Mean ± standard deviation, minimal detectable change at 95% confidence (MDC_95_), intraclass correlation coefficient (ICC), and signed and absolute relative errors (SRE and ARE, respectively) values are provided. CI: confidence interval. *: significantly different from 0 at *p* < 0.05.

**Both Legs**						
**Gait Parameters**	**GAITRite**	**NeuroSkin**	**MDC_95_**	**ICC (3,1)** **(95% CI)**	**SRE** **(95% CI)**	**ARE** **(95% CI)**
**Speed** (cm/s)	123.9 ± 18.2	124.8 ± 18.5	3.41	0.99 [0.96–1]	0.8% [−0.5%, 1.7%]	1.4% [0.8–2.3%]
**Cadence** (steps/min)	104.1 ± 9.1	103.8 ± 8.8	0.96	1 [0.98–1]	−0.3% [−0.7%, 0.1%]	0.6% [0.4–0.9%]
**Stride time** (seconds)	1.150 ± 0.1	1.156 ± 0.1	0.013	1 [0.98–1]	0.5% * [0.1%, 1.0%]	0.6% [0.4–1.2%]
**Stride length** (cm)	143.14 ± 13.87	142.73 ± 14.51	3.13	0.99 [0.95–1]	−0.3% [−1.3%, 0.7%]	1.2% [0.6–1.9%]
**Step time** (seconds)	0.581 ± 0.05	0.585 ± 0.05	0.007	1 [0.97–1]	0.6% * [0.2%, 1.3%]	0.6% [0.2–1.3%]
**Step length** (cm)	71.31 ± 7.01	71.15 ± 7.11	1.33	0.99 [0.96–1]	−0.2% [−1.3%, 0.4%]	1% [0.6–1.8%]
**Swing time** (seconds)	0.483 ± 0.04	0.42 ± 0.04	0.03	0.87 [0.55–0.97]	−1.6% [−4.5%, 1.0%]	3.9% [2.4–5.7%]
**Stance time** (seconds)	0.723 ± 0.07	0.738 ± 0.07	0.03	0.94 [0.71–0.99]	2.1% * [0.4%, 4.0%]	2.7% [1.4–4.3%]

**Table 3:** Descriptive statistics of data analyzed by leg. Mean ± standard deviation, ICC, and ARE values are provided. CI: confidence interval.

Left Leg					Right Leg				
Gait Parameters	GAITRite	NeuroSkin	ICC (3,1) (95% CI)	ARE (95% CI)		GAITRite	NeuroSkin	ICC (3,1) (95% CI)	ARE (95% CI)
**Stride time** (seconds)	1.15 ± 0.11	1.16 ± 0.11	**0.98** [0.9–0.99]	1.6% [0.7–2%5]		1.15 ± 0.1	1.15 ± 0.1	**0.96** [0.82–0.99]	1.6% [0.8–3.8%]
**Stride length** (cm)	143.25 ± 14.22	142.95 ± 14.9	**0.98** [0.9–1]	1.7% [1.3–2.6%]		143.03 ± 14.3	142.51 ± 14.93	**0.99** [0.95–1]	1.3% [0.8–2.1%]
**Step time** (seconds)	0.58 ± 0.05	0.59 ± 0.05	**0.96** [0.83–0.99]	2.4% [1.3–3.6%]		0.58 ± 0.05	0.58 ± 0.06	**0.96** [0.85–0.99]	2.4% [1.6–3.6%]
**Step length** (cm)	71.08 ± 7.86	70.51 ± 8.06	**0.98** [0.93–1]	1.4% [0.7–3.1%]		71.54 ± 6.51	71.79 ± 6.44	**0.98** [0.92–1]	1.6% [1.1–2.4%]
**Swing time** (seconds)	0.43 ± 0.04	0.42 ± 0.05	**0.8** [0.37–0.95]	4.7% [2.5–7.9%]		0.43 ± 0.04	0.42 ± 0.01	**0.79** [0.32–0.95]	4.9% [3.2–6.6%]
**Stance time** (seconds)	0.72 ± 0.08	0.74 ± 0.07	**0.91** [0.61–0.95]	3.3% [2–5.5%]		0.72 ± 0.07	0.73 ± 0.07	**0.86** [0.53–0.97]	4.3% [2.9–6.8%]

## Data Availability

Data available on request due to restrictions (The data are not publicly available due to privacy.).

## Abbreviations

The following abbreviations are used in this manuscript:

HS	Heel strike
TO	Toe off
ICC	Intraclass correlation coefficient
QGA	Quantitative gait analysis
MFES	Multi-channel functional electrical stimulation
IMU	Inertial measurement unit
GDPR	General data protection regulation
CI	Confidence interval
ARE	Absolute relative error
BCa	Bias-corrected and accelerated bootstrap
MDC	Minimal detectable changes
